# Oncolytic Replication of *E1b*-Deleted Adenoviruses

**DOI:** 10.3390/v7112905

**Published:** 2015-11-06

**Authors:** Pei-Hsin Cheng, Stephen L. Wechman, Kelly M. McMasters, Heshan Sam Zhou

**Affiliations:** 1 Department of Surgery, St. Jude Children’s Research Hospital, Memphis, TN 38105, USA; paisin.paisin@gmail.com; 2 Department of Pharmacology and Toxicology, University of Louisville School of Medicine, Louisville, KY 40292, USA; slwech01@exchange.louisville.edu (S.L.W.); mcmasters@louisville.edu (K.M.M.); 3 Department of Surgery, University of Louisville School of Medicine, Louisville, KY 40292, USA; 4 James Graham Brown Cancer Center, University of Louisville School of Medicine, Louisville, KY 40292, USA; 5 Department of Microbiology and Immunology, University of Louisville School of Medicine, Louisville, KY 40292, USA

**Keywords:** adenovirus, virotherapy, E1B, cell cycle, cancer selectivity, cyclin E

## Abstract

Various viruses have been studied and developed for oncolytic virotherapies. In virotherapy, a relatively small amount of viruses used in an intratumoral injection preferentially replicate in and lyse cancer cells, leading to the release of amplified viral particles that spread the infection to the surrounding tumor cells and reduce the tumor mass. Adenoviruses (Ads) are most commonly used for oncolytic virotherapy due to their infection efficacy, high titer production, safety, easy genetic modification, and well-studied replication characteristics. Ads with deletion of *E1b55K* preferentially replicate in and destroy cancer cells and have been used in multiple clinical trials. H101, one of the *E1b55K*-deleted Ads, has been used for the treatment of late-stage cancers as the first approved virotherapy agent. However, the mechanism of selective replication of *E1b*-deleted Ads in cancer cells is still not well characterized. This review will focus on three potential molecular mechanisms of oncolytic replication of *E1b55K*-deleted Ads. These mechanisms are based upon the functions of the viral E1B55K protein that are associated with p53 inhibition, late viral mRNA export, and cell cycle disruption.

## 1. Introduction

Cancer is the most common cause of death in the world [1]. The limitations of currently available therapies demand development of more potent cancer treatment options. Oncolytic virotherapy represents a fast-growing therapeutic platform for cancer treatment [2,3]. The therapeutic effects emanate from a relatively small amount of viruses that preferentially replicate in and lyse cancer cells, followed by a localized spread of viral infection to the surrounding tumor cells, ultimately leading to reduction of the tumor mass [4]. Current oncolytic viruses include adenovirus (Ad), herpes simplex virus, measles virus, newcastle disease virus, reovirus, parovirus, poliovirus, seneca valley virus, retrovirus, vaccinia, and vesicular stomatitis virus, which have been tested in numerous preclinical or clinical settings [5]. Although many viruses have been developed, Ads are still the most commonly used for oncolytic virotherapy due to their infection efficacy, high titer production, safety, easy genetic modification, and well-studied replication characteristics [6]. This review will focus on molecular mechanisms of the selective replication of *E1b*-deleted Ads in cancer cells.

## 2. Oncolytic Adenoviruses

Human Ads, as a common cause of respiratory tract diseases, are non-enveloped DNA viruses that can infect cells at many different cell cycle stages without integrating into host cellular chromosomes [7]. Ad serotype 5 (group C) has been modified and widely used in oncolytic virotherapy due to its favorable safety profile and only causing negligible flu-like symptoms, such as fever, myalgia, asthenia, and chills [8]. The viral genome is composed of a linear, double-stranded DNA of approximately 36 kb and can be divided into *early* (*E*) and *late* (*L*) genes [7,9]. The *E1* genes, including *E1a* and *E1b*, produce critical proteins for the initiation and regulation of several viral and cellular genes. The *E2* genes encode three different proteins which function directly in viral DNA replication. The *E3* genes encode proteins that can modulate infected host immune responses. Products of the *E4* genes encode proteins of diverse functions, such as regulating viral gene transcription, translation, mRNA nuclear export, and apoptosis pathways. The *L* genes of Ads encode structural proteins for packaging the viral genome into virion particles in the final stages of virus replication.

Ad *E1b* gene encodes two major polypeptides of 55,000 kDa (55K) and 19,000 kDa (19K), both required for transformation of rodent cells by viral infection and DNA transfection [10,11]. The E1B55K protein protects the infected cells from the E1A-induced p53 effects. The E1B55K protein also modulates transport or cytoplasmic stabilization of viral and host cell mRNA [12]. The Ad E1B19K protein is the putative B-cell lymphoma 2 protein (Bcl-2) functional homolog and a strong apoptotic inhibitor [13,14,15]. E1B19K prevents E1A-induced apoptosis by interfering with the actions of the pro-apoptotic proteins Bak and Bax [16]. Through the action of these E1b-encoded proteins, premature cell death is believed to be prevented and viral replication is maximized.

**Figure 1:**
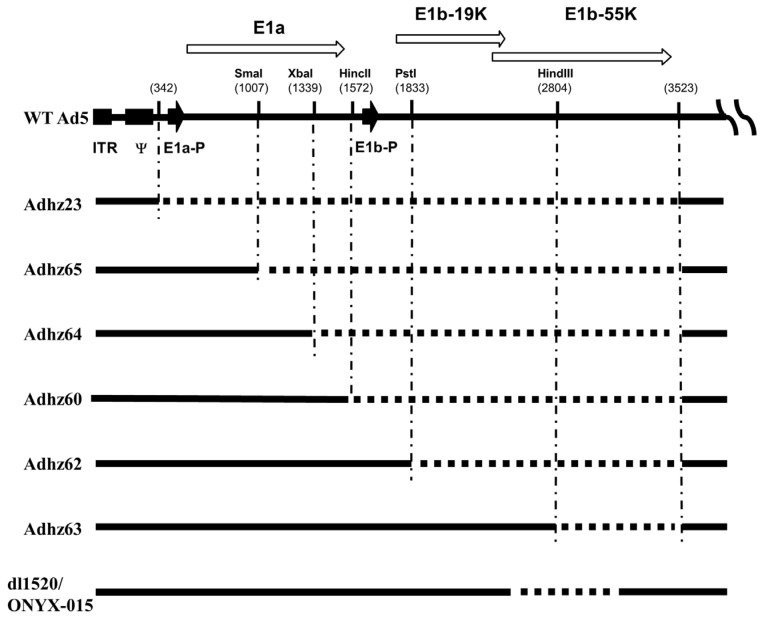
Depiction of the human Ad5 left end with various deletions in *E1* genes. All the nucleotide coordinates refer to Ad5 (Genbank Accession No M73260). The wild-type Ad5 with the *E1a* and *E1b* genes and their endogenous promoters is shown at the top. The left inverted terminal repeat (ITR), packaging signal (Ψ), the *E1a* and *E1b* promoters (E1a-P and E1b-P), and the open reading frames are indicated. E1B19K and E1B55K have a small overlap. The solid lines represent Ad regions in these viruses, and the dashed lines represent the deleted regions. The restriction enzyme and the cleaving sites used to delete the *E1* region are shown. Ad dl1520 carries native E1A promoter (E1A-P) to drive E1A expression and an 827-bp deletion with a point mutation to generate a premature stop codon in the E1B55K coding region.

We made a series of deletions in the E1 region, ranging from partial to total deletion of the *E1* gene (Figure 1) [17]. We have showed that a virus with deletion of both *E1b55K* and *E1b19K* induced more profound apoptosis in comparison to wild-type Ad and viruses with a mutation only in *E1b55K* [17]. However, the virus deleted for both *E1b55K* and *E1b19K* still can replicate in the majority of the infected cancer cells. Therefore, the p53 inactivation and apoptosis inhibition by E1B55K and E1B19K are not critically necessary in virus replication, implying that the E1B proteins may have other roles. An approach based on a large-scale gene array was applied to analyze the expression of cellular genes affected by the *E1b* gene [18]. We identified a total of 345 genes with expression changes of two-fold or greater affected by wild-type Ad compared with its E1B-deleted counterpart. Thus, the E1B proteins affect the expression of a diverse range of genes, including *CDC25A* and *cyclin E*, which are important in cell cycle regulation [18].

The Ad infection begins by the binding of the knob domain on the Ad fiber to a specific coxsackievirus and Ad receptor (CAR) located on the cell surface. The fiber and penton base of Ads have the functions of attachment and internalization, respectively, that facilitate the entry of the virus into the host cells by endocytosis [19]. The low-pH environment of the endosome allows the viral capsid to disassociate, resulting in the release of virions from the endosome [20,21,22]. By cooperating with microtubules, virions are then transported to the nuclear pore complex where the virions disassemble and the double-stranded viral DNA is released to directly enter the nucleus.

The replication of Ads relies on utilizing the cellular components as building blocks. The replication cycle of Ads is divided into two phases based on the onset of viral gene expression [7]. The early phase includes the expression of a set of viral genes to promote cellular entry into an S-like phase, block apoptosis, and prevent cellular immune responses. After the expression of viral early genes that control critical cellular components and regulate viral and host gene expression, Ads usurp the cellular replication machinery, viral DNA replicates. Viral gene expression then transitions to the late phase in which the structural proteins are synthesized. Once Ads have finished the DNA replication and synthesis of structural proteins, viral assembly takes place and produces virions, leading to significant amplification of the Ads with up to 10,000 viral particles per cell. Viral replication subsequently causes cell lysis and the release of progeny virions [7,23].

The idea of treating cancers with Ads began in the 1950s [5,24]. Wild-type Ad5 was injected in 30 patients with epidermoid cervical carcinomas and 65% of the virus injections induced necrosis in the tumors without causing severe toxicity. While producing some promising results, the long-term safety concerns, limited final therapeutic efficacy, and tumor recurrence after the treatment prevented further development of this approach until recently.

Ad dl1520 (ONYX-015) is a gene-attenuated oncolytic virus with an 827-bp deletion and a point mutation in the *E1b* region which generates a premature stop codon to prevent the expression of its E1B55K protein (Figure 1) [10,25,26]. dl1520 showed potent and promising oncolytic efficacy in several preclinical studies and became the first oncolytic Ad used in clinical trials in the United States since 1996; approximately 200–300 cancer patients were treated with various routes of administration in more than 10 clinical trials (from phase I to II) [26,27]. However, during a phase III clinical trial in 2003, when treatment with dl1520 was combined with chemotherapy in patients with head and neck squamous cell carcinoma, the trial was suspended due to limited therapeutic potential in metastatic cancer patients [27].

H101 (Oncorine), another Ad vector with the same deletion of the *E1b55k* region as dl1520, was generated by the Chinese company Sunway Biotech [27,28]. It is believed that H101 is similar to dl1502 and has the same oncolytic properties. The phase I clinical trial with H101 as a single therapeutic agent began in 2000 and the phase II and III trials of H101 in combination with chemotherapy started from 2001 to 2004 in China. Overall, the clinical tolerability and responses of H101 treatment have been very promising [27,28]. The rights to dl1520 were bought by Sunway Biotech which has completed Phase III clinical studies with H101. More than 600 cancer patients have safely received the treatment with H101 in clinical trials [27,29]. The company reported a 79% positive response rate for H101 plus chemotherapy as compared to 40% for chemotherapy alone [30]. Considering the impact of these results, the Chinese State Food and Drug Administration approved H101 for use in combination with chemotherapy for the treatment of late-stage cancers. Thereafter, Sunway Biotech has marketed H101 as the first approved virotherapy agent for clinical applications [30,31]. Besides H101, this company has also developed other genetically modified oncolytic adenoviruses, H102 and H103, for cancer treatment [32]. In H102, expression of the Ad *E1a* gene is driven by the alpha-fetoprotein promoter which is highly activated in primary hepatocellular carcinoma, but not in normal cells. Thus, H102 may selectively express the viral *E1a* gene in liver cancer, leading to specifically oncolytic replication. H102 is currently in the preclinical stage of development. H103 is an oncolytic Ad carrying a tumor antigen known as the *heat shock protein (HSP) 70* gene which can stimulate an antitumor immune response while the virus selectively replicates within tumor cells. A phase I clinical trial of intratumoral injections of H103 has been conducted in a total of 27 patients with advanced-stage solid tumors [29]. An 11.1% objective response (cases with complete response plus partial response) to H103-injected tumors and a 48.1% clinical benefit rate (cases with complete response plus partial response, minor response, and maintenance of stable disease) was reported.

## 3. Selective Replication of *E1b55K*-Deleted Ad in Cancer Cells

The antitumor efficacy of *E1b55K*-deleted Ad directly relies on virus-mediated tumor cell lysis by selective viral replication [33]. During the process of virus replication, the expression of virus proteins such as E1A [14], E3-11.6K [34], and E4ORF4 [35] can cause cytotoxicity to cells by inducing apoptosis. Recent studies also showed that autophagy participates in the oncolytic Ad replication and virus-induced oncolysis [36,37]. After virus infection and replication, the induction of antitumoral immunity by recruiting natural killer cells, antibodies, or tumor-specific cytotoxic T lymphocytes (CTLs) to virus-infected tumor cells may also enhance the antitumoral outcome [38,39]. Undoubtedly, the selective replication of oncolytic Ads is the front line of the oncolysis process. Yet, even though *E1b55K*-deleted oncolytic Ads have been used in many clinical trials and are marketed in China, the mechanism(s) enabling this selective oncolytic replication still remains controversial. The following sections summarize three potential mechanisms proposed until now.

## 4. Cancer Selectivity of *E1b55K*-Deleted Ads Based on p53 Deficiency

The original hypothesis was that *E1b55K*-deleted oncolytic Ads could only replicate in p53-deficient tumor cells, but not in normal cells with functional p53 [25,26,40]. After virus infection, Ad E1A is expressed immediately to regulate the expression of viral genes and promote cell entry into an S-like phase [41]. In response to Ad infection, host cells have developed a crucial strategy to block the virus spread by activation of p53-mediated apoptosis and cell cycle arrest [7,17,42]. E1A triggers the accumulation of the p53 protein either by activating *p53* transcription [43] or stabilizing p53 via inducing the expression of p14^ARF^ tumor suppressor (referred to as p19^ARF^ in murine cells) which binds to MDM2 protein and subsequently blocks MDM2-induced p53 degradation and transactivational silencing [44,45,46]. Consequently, high levels of p53 in infected cells lead to either cell cycle arrest or apoptosis to block viral replication and spread. In response to this, Ad has developed its ways to overcome p53-mediated apoptosis and cell cycle arrest. It was reported that Ad E1B55K prevents the E1A-induced p53 effects through at least three distinct mechanisms. First, E1B55K binds to the amino terminus of p53, and thus represses p53 transactivation [47,48]; second, E1B55K cooperates with Ad E4orf6 to proteolytically degrade p53 [49,50,51]; third, E1B55K alone can function as an E3 SUMO1-p53 ligase, eventually leading to p53 polyubiquitinylation and proteasomal degradation [52]. It was believed that E1B55K counteracts the p53-dependent apoptosis induced by E1A and prevents premature cell death, resulting in efficient viral replication in normal cells [47,48]. Without the E1B55K protein, Ads were presumably unable to counteract E1A-induced p53 accumulation and thus failed to replicate in cells with functional p53 protein. Deficiency of p53 occurs frequently in many types of human cancers due to *p53* gene deletion or mutation [53,54]. It was proposed that since many cancer cells lack functional p53 protein, this E1B55K function is not as important as it is in normal cells; thus dl1520 could selectively replicate in cancer cells with dysfunctional p53 pathways [25,26].

While some reports have supported the hypothesis that *E1b55K*-deleted Ads selectively kill cancer cells with p53 deficiency, the original proposal has been challenged by several studies. Reports have shown that *E1b55K*-deleted Ads are able to replicate in and kill cancer cells with wild-type p53 as efficiently as in cancer cells with p53 deficiency [55,56,57,58,59]. To resolve this paradox, it was proposed that either p53 deficiency or p14^ARF^ deficiency might determine the cancer selectivity of *E1b55K*-deleted Ads [60,61]. In cancer cells with wild-type p53, loss of the functional p14^ARF^ would neutralize MDM2-mediated p53 degradation and was considered to prevent p53 from its normal functions. Thus, p14^ARF^ deficiency in cancer cells was indicated as the alternative molecular mechanism to allow dl1520 replication in these cancer cells with wild-type p53. However, experimental data contradicted this mechanism by showing that the replication of dl1520 is not controlled by the p53 or p14^ARF^ status in several cancer cell lines [62]. Also, it has been reported that, in some cell lines, p53 can promote the viral lytic cycle and may be required for productive virus replication and late viral gene expression by cooperating with E1A to enhance transcription at the major late gene promoter of the viral genome [63,64]. Further studies also revealed that the accumulation of p53 induced by *E1b55K*-deleted or *E1b55K*-mutated Ads can neither efficiently induce apoptosis nor activate the transcription of downstream p53-responsive genes in Ad-infected primary cells [65,66]. Moreover, in cells that are infected with *E1b55K*-deleted Ads, *E1b19K*, as a homologue of BCL-2 family members, can also function as an inhibitor to E1A protein-induced apoptosis [17]. Even if E1A-induced apoptosis occurs in cancer cells infected with Ads with deletion of the entire *E1b* gene, it does not prevent the replication of the *E1b*-deleted oncolytic Ads in the majority of cells [17]. Thus, the p53 deficiency in cancer cells seems unlikely to be the determinant of oncolytic selectivity of *E1b55K*-deleted Ads and blocking p53 activity may not be the major requirement for viral replication. Thus far, none of the comprehensive research has been reported to provide new data to further support the p53 hypothesis.

## 5. Cancer Selectivity of *E1b55K*-Deleted Ads Based on Late Viral mRNA Export

It was proposed that cancer cells can provide the functions of the E1B55K protein for late viral RNA export [66]. During the virus replication process, the amounts of late viral mRNAs are raised in the cytoplasm for translation [7]. E1B55K functions in a complex with the viral E4orf6 protein to direct the switch from host to viral protein synthesis by promoting the preferential nuclear export of the late viral mRNAs to the cytoplasm for viral protein synthesis [12,66,67,68,69,70]. Several lines of evidence support this hypothesis. The E1B55K/E4orf6 complex is directly involved in the selective nuclear export of late viral mRNAs through active nucleocytoplasmic shuttling via the exportin CRM1 [7,67,71] or through its E3 ubiquitin-protein ligase activity [70,72,73]. Since *E1b55K*-deleted oncolytic Ads lack E1B55K-mediated late viral mRNA export from the nucleus in primary cells, the viruses fail to efficiently replicate in those normal cells [66]. Permissive cancer cells, however, have a propensity to support the nuclear export of viral late mRNAs in the absence of E1B55K while normal cells do not, allowing the oncolytic replication of *E1b55K*-deleted Ads. However, the molecular mechanism(s) of either selective nucleocytoplasmic transport mediated by the E1B55K/E4orf6 complex or the assembly and activity of the E3 ubiquitin-protein ligase associated with E1B55K/E4orf6 complex has not been fully understood [72].

There is a report indicating that either restoring viral L4 100K protein expression or inducing heat shock responses can partially rescue *E1b55K*-deleted Ad replication in refractory cancer cells [74]. In contrast to permissive cancer cell lines, refractory cancer cell lines fail to offer preferential nuclear export and translation of viral late mRNAs including Ad L4 100K mRNA in the absence of E1B55K [74]. Ad L4 100K is known to be involved in host protein shutoff and promotes preferential nuclear export and translation of viral late mRNAs [75,76,77]. As a consequence of the absence of E1B55K, the lack of L4 100K expression in refractory cancer cells leads to the poor oncolytic Ad replication; however, ectopic expression of L4 100K enhanced the expression of late viral proteins in refractory cancer cells [74]. In the same report, the authors also suggested that heat shock proteins (Hsp) overexpressed in various human cancers may be an important molecular mechanism to support the differential transport of late viral mRNA export. The cellular responses to heat shock resemble the late stages of Ad infection with the inhibited translation of cellular mRNAs and the preferential translation of heat shock mRNAs. Also, it has been shown that the 5′UTR of mammalian Hsp70 and late Ad mRNAs share structural homology, which may contribute to both selective translation and nuclear export through a common mechanism [78]. Thus, the induction of heat shock responses by physical or pharmacological means could selectively rescue the export of late viral mRNAs in refractory cancer cells, rendering them once again permissive to *E1b55K*-deleted Ad replication.

Although expression of L4 100K and inducing heat shock responses may participate in determining the cancer selectivity of *E1b55K*-deleted Ads on late viral mRNA export, evidence has also shown neither expressing L4 100K nor inducing heat shock responses could completely restore *E1b55K*-deleted Ad replication to the level of wild-type Ad in a majority of cancer cell lines [74].

Since ectopic expression of L4 100K and inducing heat shock responses cannot recover wild-type replication capacity in the absence of E1B55K, the importance of E1B55K for selective oncolytic Ad replication cannot be ignored. The exact mechanics of the interaction of E1B55K, L4 100K, and heat shock responses to regulate mammalian and late viral mRNA export which leads to cancer selectivity of *E1b55K*-deleted Ads are still required for further studies. Studies conducted by Goodrum and Ornelles (1999) have shown that the E1B55K function in late viral mRNA export is not correlated with the restricted replication of *E1b55K*-deleted Ads in cells under G_0_/G_1_ phase [69]. Gonzalez *et al.* (2006) indicated that it appears overly simplistic to generalize that the cancer selectivity of *E1b55K*-deleted Ads relies on the efficiency of viral late mRNA export [79]. E1B55K has many more functions than regulating the differential export of mRNAs that are important for Ad replication and, therefore, the outcome of a complex interaction between E1B55K and multiple cellular components should be considered holistically in determining the cancer selectivity mechanism.

## 6. Cancer Selectivity of *E1b55K*-Deleted Ads Based on Cyclin E or Cell Cycle Dysregulation

The study of Goodrum and Ornelles has shown that unlike wild-type Ad, which is able to efficiently produce viral progeny regardless of the cell cycle stages, the replication of *E1b55K*-deleted Ads is restricted in HeLa cells under the G_0_/G_1_ phase, but is less restricted under the S-phase [69]. However, the total amount of cytoplasmic late viral mRNA was found to be greater in cells infected during the G_1_ phase than that in cells infected during the S-phase with either the wild-type or *E1b55K*-deleted Ads. A further study suggested that the E1B55K protein has a property to enable the virus to overcome the growth restrictions to its replication imposed by the cell cycle stage [80]. In addition, viral *E1b* products affected the expression of numerous genes involved in cell cycle regulation based on a study with large-scale gene arrays [18]. The levels of some key regulators of cell cycle progression, including cyclin E and CDC25A, were significantly increased by E1B. Thus, E1B55K may have a novel function in the induction of cyclin E expression and cell cycle regulation that may be critical for virus replication.

Cyclin E is well characterized as a critical cell cycle protein to promote G_1_/S phase transition [81,82] in either a cyclin-dependent kinase 2 (CDK2)-dependent [83] or CDK2-independent manner [84,85]. The expression of cyclin E is strictly controlled in normal cells, while the overexpression or deregulation of cyclin E is related to tumorigenesis [86,87,88,89]. *Cyclin E* gene amplification [90], overexpression of cyclin E mRNA or protein levels [88,91], decrease of cyclin E turnover [92], together with the presence of more active forms of cyclin E [93,94,95], have been reported in many types of cancers, such as breast, gastrointestinal, lung, and skin cancers. Constitutive overexpression of cyclin E can induce chromosome instability and impair normal cell cycle progression [96,97].

**Figure 2:**
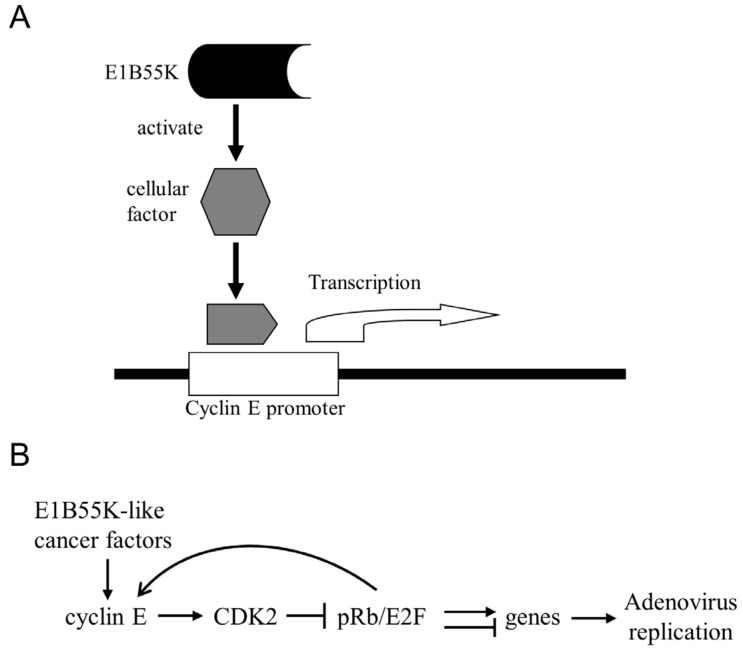
Cyclin E upregulation in cancer cells supports oncolytic replication of *E1b55K*-deleted Ads. (**A**) In normal cells, Ad E1B55K functions to enhance cyclin E expression for virus replication. E1B55K may target cellular factors which, in turn, activate the cyclin E promoter for induction of the gene expression. Without E1B55K-induced cyclin E expression, replication of *E1b55K*-deleted Ads is repressed in normal cells; (**B**) In cancer cells, the cellular factors may already be activated or there are E1B55K-like factors to relax cyclin E regulation. Thus, E1B55K protein is not required to induce cyclin E expression in cancer cells. Cyclin E induced by Ad infection binds to and activates CDK2, which subsequently phosphorylates the transcriptional repressor retinoblastoma protein (pRb), leading to regulative expression of multiple genes (including the feedback upregulation of cyclin E) to provide a suitable cellular environment for oncolytic replication.

The replication of *E1b55K*-deleted Ads is restricted in normal cells, which may be due to the failure of efficient induction of cyclin E expression because of the deletion. We have shown that the induction of cyclin E is required for Ad replication and is correlated with the oncolytic selectivity of *E1B55K*-deleted Ad [18,59]. We also have shown that Ad-induced cyclin E highly interacts with CDK2 in cancer cells, which is a critical molecular step in Ad replication. The cyclin E protein induced by Ad infection directly interacted with CDK2 and formed the cyclin E/CDK2 complex, leading to the specifically increased phosphorylation of CDK2 at T160 and of the retinoblastoma protein (pRb) at S612 [98]. The inhibition of CDK2 expression with the CDK2 siRNA or the CDK2 chemical inhibitor roscovitine repressed phosphorylation on CDK2 and pRb and decreased viral replication. These results suggest that Ad-induced cyclin E activates CDK2 by phosphorylating at T160, which then specifically introduces pRb phosphorylation at the S612 site. The pRb phosphorylation caused by cyclin E/CDK2 may lead to the regulation of multiple cellular and viral genes for productive Ad replication. Cancer cells with relaxed cyclin E regulation allow *E1b55K*-deleted Ads to induce cyclin E expression in the absence of the E1B55K function, leading to preferential oncolytic replication [98]. We thus proposed that cyclin E overexpression or dysregulation in cancer cells may be a molecular basis of the oncolytic selectivity of *E1b55K*-deleted Ads (Figure 2).

## 7. Conclusions and Future Perspectives

Ads with the deletion of *E1b55K* preferentially replicate in cancer cells and have been used in oncolytic virotherapies. However, many clinical studies have demonstrated the limited therapeutic effects in metastatic cancer patients. By far, oncolytic Ads have only shown some therapeutic benefits in the treatments of localized tumors. At least three mechanisms have been proposed so far. The original hypothesis was that, since many cancer cells lack functional p53 protein or its pathways, the E1B55K function of inhibiting p53 is not as important as it is in normal cells, and thus, *E1b55K*-deleted Ads could selectively replicate in cancer cells with p53 deficiency or dysfunctional pathways. The second mechanism is that cancer cells have a propensity to support the nuclear export of viral late mRNAs in the absence of the E1B55K protein, and thus allow the oncolytic replication of *E1b55K*-deleted Ads. The third mechanism is that cancer cells with dysregulated cyclin E allow *E1b55K*-deleted Ads to induce cyclin E expression, leading to preferential oncolytic replication. Understanding the molecular mechanisms underlining the cancer-selective replication of *E1b55K*-deleted Ads will guide us to develop new oncolytic vectors and therapeutic strategies. As the selective replication of *E1b55k*-deleted Ads is dependent on cyclin E dysregulation in cancer cells, better virotherapeutic efficacy may be expected when this kind of vector is used to target aggressively growing tumor cells with cyclin E overexpression. We have reported that the activity of cyclin E promoter is augmented in cancer cells during Ad infection [59,99]. The unique property of the cyclin E promoter has been applied in the construction of a new oncolytic Ad vector. The cyclin E promoter is used to drive E1A expression in a vector which has shown selective and efficient antitumor effects *in vitro* and *in vivo* [36,100,101]. Future vectors may combine this strategy with the other strategies, such as genetic modification on viral fibers or arming the virus with cytokine or anticancer genes to create the new generation of tumor-specific adenoviruses.

Ad E1B55K protein has multiple functions and interacts with various cellular components, which are still not fully understood. The oncolytic replication of *E1b55K*-deleted Ads may be determined by several factors in cancer cells; some factors may be more important than others, depending on different types of tumors or cancer cells. To improve oncolytic Ad therapies for localized tumors, it is important to increase virus release from the initially targeted cells and promote Ad to spread the infection to the entire tumor mass. By further exploring the questions left in current mechanisms, strategies can be developed to overcome the limitations and ultimately build up new, powerful oncolytic Ads for virotherapy.
